# Medicinal Cannabis and Central Nervous System Disorders

**DOI:** 10.3389/fphar.2022.881810

**Published:** 2022-04-21

**Authors:** Yuma T. Ortiz, Lance R. McMahon, Jenny L. Wilkerson

**Affiliations:** ^1^ Department of Pharmacodynamics, College of Pharmacy, University of Florida, Gainesville, FL, United States; ^2^ Department of Pharmaceutical Sciences, School of Pharmacy, Texas Tech University Health Sciences Center, Amarillo, TX, United States

**Keywords:** cannabinoid 1 receptor, cannabinoid 2 receptor, serotonin 1a receptor, clinical research, addiction, pain, neurodegeneration, anxiety

## Abstract

Cannabinoids, including those found in cannabis, have shown promise as potential therapeutics for numerous health issues, including pathological pain and diseases that produce an impact on neurological processing and function. Thus, cannabis use for medicinal purposes has become accepted by a growing majority. However, clinical trials yielding satisfactory endpoints and unequivocal proof that medicinal cannabis should be considered a frontline therapeutic for most examined central nervous system indications remains largely elusive. Although cannabis contains over 100 + compounds, most preclinical and clinical research with well-controlled dosing and delivery methods utilize the various formulations of Δ9-tetrahydrocannabinol (THC) and cannabidiol (CBD), the two most abundant compounds in cannabis. These controlled dosing and delivery methods are in stark contrast to most clinical studies using whole plant cannabis products, as few clinical studies using whole plant cannabis profile the exact composition, including percentages of all compounds present within the studied product. This review will examine both preclinical and clinical evidence that supports or refutes the therapeutic utility of medicinal cannabis for the treatment of pathological pain, neurodegeneration, substance use disorders, as well as anxiety-related disorders. We will predominately focus on purified THC and CBD, as well as other compounds isolated from cannabis for the aforementioned reasons but will also include discussion over those studies where whole plant cannabis has been used. In this review we also consider the current challenges associated with the advancement of medicinal cannabis and its derived potential therapeutics into clinical applications.

## 1 Introduction

Interest in cannabinoids has continued to grow as they steadily show increased potential as therapeutics for treating a diverse range of diseases and illnesses. Therapeutic actions of these cannabinoids are in part the result of two identified cannabinoid receptors, both of which are G protein-coupled receptors. Cannabinoid 1 receptors (CB_1_R) appear in high densities among presynaptic neurons within the central nervous system (CNS), particularly among GABAergic interneurons, and peripheral neurons as well as on astrocytes and oligodendrocytes (Huang et al., 2001; Katona et al., 2001; Ohno-Shosaku et al., 2001; Szabo et al., 2005). The behavioral effects of cannabinoid consumption, termed as “cannabimimetic” behavioral effects, are mediated by neuronal CB_1_R. Such cannabimimetic effects include acute antinociception, decreased locomotion, catalepsy, and hypothermia (Little et al., 1988; Ledent et al., 1999; Grim et al., 2016). CB_1_R are also associated with anti-inflammatory mechanisms, contributing to therapeutic prospects of CB_1_R agonists (Richardson et al., 1998; Kraus et al., 2009; Newton et al., 2009). CB2 receptors (CB_2_R) are expressed by immune cells including microglia, astrocytes, oligodendrocytes (Munro et al., 1993;Galiegue et al., 1995; Schatz et al., 1997; Carayon et al., 1998), and discrete neuronal populations (Onaivi et al., 2008a) within the brainstem (Van Sickle et al., 2005), and the hippocampus (Stempel et al., 2016). Unlike CB_1_R agonism, CB_2_R agonism does not result in the cannabimimetic effects observed with CB_1_R agonists whilst still producing anti-inflammatory signaling cascades (Rahn et al., 2011). Despite the current, vast library of synthetic cannabinoid ligands generated, clinical research has extensively utilized variations of Δ9-tetrahydrocannabinol (THC) and cannabidiol (CBD) due to their well-controlled dosing and delivery methods. Within this review, we report on the use of THC (a non-selective CB_1_R/CB_2_R agonist) and its clinically approved synthetic formulations, dronabinol and nabilone, THC/CBD formulations as nabiximols and medicinal cannabis, and CBD (limited agonism at CB_1_R/CB_2_R) and its clinically approved formulation, Epidiolex. Not discussed at length within this review, it should be noted that medicinal cannabis contains over 100 different compounds, including acid phytocannabinoids, cannabigerol, as well as cannabis-related terpenoids (Russo, 2018). Each of these cannabis compounds has its own pharmacology which can include activity at receptors not discussed within this review, and these compounds may modify resultant THC, CBD activity as well as overall medicinal cannabis therapeutic potency.

**FIGURE 1:**
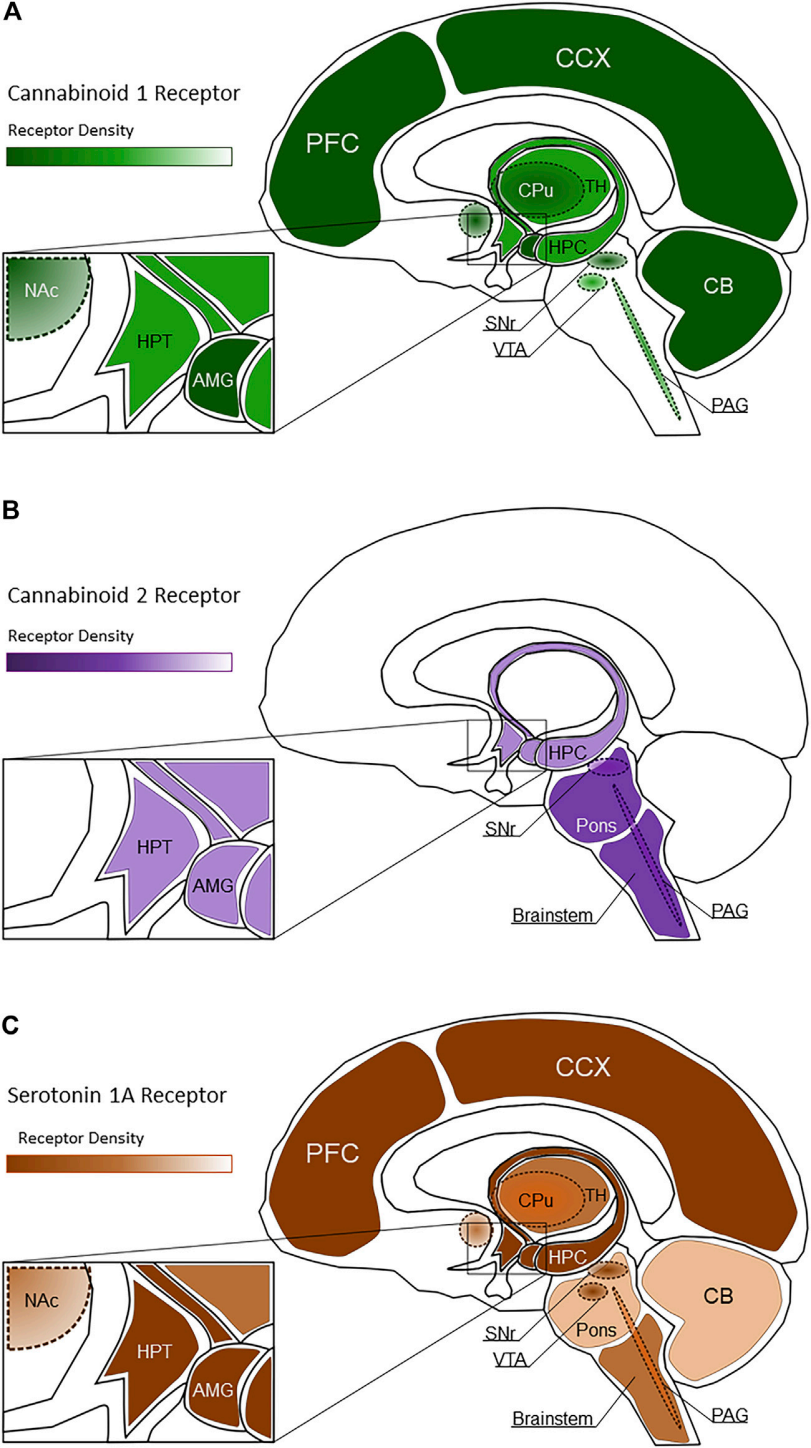
Spatial distribution of CB_1_R [**(A)**, green shading], CB_2_R [**(B)**, purple shading], and 5-HT1A receptors [**(C)**, brown shading] within healthy brain regions. Lighter shaded regions represent low receptor density while darker shaded regions represent high receptor density. In these images, PFC, Prefrontal Cortex; CCX, Cerebral Cortex; CB, Cerebellum; CPu, Caudate Putamen; HPC, Hippocampus; TH, Thalamus; HPT, Hypothalamus; Nac, Nucleus Accumbens; SNr, Substantia Nigra pars compacta; VTA, Ventral Tegmental Area; PAG, Periaqueductal gray; AMG, Amygdala.

The therapeutic actions of CBD are generally attributed to non-CB_1_R/ CB_2_R activity, including partial agonist activity at the 5-HT1A receptor, although potential endocannabinoid modulatory effects of CBD cannot be eliminated (De Gregorio et al., 2019; King et al., 2017). Serotonin 5-HT1A receptors are G protein-coupled receptors located on presynaptic serotonergic neurons and postsynaptic non-serotonergic neurons, astrocytes, oligodendrocytes, and microglia, with a high density of distribution within limbic brain areas (Barnes & Sharp, 1999; Riad et al., 1991, 2000). Additionally, 5-HT1A receptors are also expressed within primary afferent neurons and their peripheral terminals (Björk et al., 1992; Granados-Soto et al., 2010; Laporte et al., 1995; Perrin et al., 2011). Figure 1shows the spatial distribution of CB_1_R, CB_2_R, and 5-HT1A receptors within the brain. Agonism of 5-HT1A receptors has been shown to inhibit nociception (Gjerstad et al., 1996; Bardin et al., 2003; Haleem & Nawaz, 2017), exhibit neuroprotective effects (Miyazaki et al., 2013; Isooka et al., 2020; Kikuoka et al., 2020), and alleviate the severity of several anxiety disorders (Sussman and Joffe, 1998; Campos and Guimarães, 2008).

The diverse physiological activity resultant of these cannabinoids indicates a wide breadth of potential therapeutic indications. As medicinal cannabis related clinical research has predominately focused on CNS-related diseases, such as neurodegeneration and neurological disorders, pain, substance use disorders, and anxiety disorders, this review will first examine evidence that supports or refutes the therapeutic utility of cannabinoids for the treatment of neurodegenerative disease, pain, mood disorders, and substance use disorders. Table 1 summarizes these clinical studies. Not discussed at length within this review, medical cannabis has also shown promise in treating other CNS-related diseases, such as brain tumors and gliomas (Russo, 2018). Despite continued growing interest and an increasing trove of preclinical research that exemplifies the therapeutic potential of cannabinoids, the development of viable, approved therapeutics remains elusive due to various challenges in formulation and bioavailability, efficacy, and tolerability. In this review we will address some of the challenges and considerations within the cannabinoid field that may be important in advancing such therapeutics into the clinic while presenting recent findings that provide a more up to date understanding of where the field currently lies regarding the therapeutic viability of cannabinoids.

**TABLE 1:** Cannabinoids and the clinical work done investigating them as novel therapeutics.

Compound	Safety	Clinical outcomes	References
Nabilone	No major adverse effects: minor side effects include fatigue, dizziness, anxiety, dry mouth	- Improvements among both motor and non-motor symptoms of PD.	Colwill et al., 2020; Hill et al., 2017; Kayser et al., 2020a; Levin et al., 2017; Peball et al., 2020; Ruthirakuhan et al., 2020
		- Improved motor function in MS patients. No improvement in cognitive function in MS patients. Self-reported improvements in pain measures among MS patients	
		- Minimal effect on symptoms of OCD, significant therapeutic effect observed when paired with behavioral therapy	
		- Nabilone exhibits anti-inflammatory effects in instances of AD.	
		- Failed to minimize post operative nausea and vomiting	
		- Reduced cannabis use among cannabis dependent patients, not discernable from placebo	
		- No reduction on maximal pain levels experienced by women undergoing medical abortion	
Dronabinol	No major adverse effects: side effects include euphoria, dry mouth	- Inconsistent acute analgesia observed with hydromorphone coadministration in healthy patients. No effect on chronic pain	Babalonis et al., 2019; Dunn et al., 2021; Malik et al., 2017; Schimrigk et al., 2017; University of California, Davis, 2018
		- Reduced pain intensity, though no different from placebo in alleviating neuropathic pain	
		- Reduced pain perception in patients with noncardiac chest pains	
		- Dronabinol did not enhance analgesia produced by oxycodone and increased abuse-related subjective effects	
Nabiximols	No major adverse effects: side effects include sedation, dizziness, nausea	- Slight improvements in self-reported pain evaluations in advanced cancer. Improved quality of life for secondary symptoms associated with advanced cancer	Collin et al., 2007; Fallon et al., 2017; Johnson et al., 2010; Kavia et al., 2010; Lichtman et al., 2018; Lintzeris et al., 2019; Markovà et al., 2019; Notcutt et al., 2004; Nurmikko et al., 2007; Portenoy et al., 2012; Riva et al., 2019; Rog et al., 2005; Trigo et al., 2018; Wade et al., 2004; Wade et al., 2006
		- Reduced the amount of cannabis consumed by cannabis dependent patients and reduced the number of cravings	
		- Reduced spasticity in patients with motor neuron disease and MS.	
Cannabidiol	No major adverse effects: side effects include fatigue, diarrhea	- Reduction of tremors, improved sleep, and improved emotional control in PD patients	Capano et al., 2020; Leehey et al., 2020; Xu et al., 2020
		- Chronic pain patients reduced or eliminated use of prescribed opioids when CBD is added to regimens	
		- Symptomatic relief of peripheral neuropathy of the lower extremities	
Whole Cannabis	No major adverse effects: side effects include sedation, anxiety in high THC concentrations	- Reduced reported intensity of chronic pain among patients with general improvements to anxiety and depression	Bonn-Miller et al., 2021; Haroutounian et al., 2016; Kayser et al., 2020b; O’Connell et al., 2019; Rudroff, 2020; Vigil et al., 2017; Ware et al., 2010, 2015; Zajicek et al., 2012
		- Decrease in maximum strength among those with MS and consuming medicinal cannabis. Relief of muscle stiffness observed after 12 weeks of consumption	
		- No difference in anxiolytic effects observed compared to placebo in PTSD patients	
		- Minimal acute effect on OCD associated anxiety compared to placebo	
		- Reduced consumption of prescribed opioids among patients with chronic pain. Instances of opioid cessation	

## 2 Cannabinoid Involvement in Central Nervous System Diseases and Disorders

### 2.1 Neurodegenerative and Neurological Diseases

Neurodegenerative and neurological diseases that commonly afflict those in mid to late life have steadily become a common cause of mortality worldwide as elderly populations have continued to grow. Epidemiological reviews of these neurodegenerative diseases show that associated deaths have increased within the last 25 years, having increased worldwide by more than 35% (Group GNDC, 2017).

#### 2.1.1 Alzheimer’s Disease

Alzheimer’s disease (AD) is the most common neurodegenerative disease that contributes to approximately 60–80% of all dementia cases globally (Erkkinen et al., 2018) and is characterized by the formation of β-amyloid plaques, phosphorylated tau proteins, formation of neurofibrillary tangles, glial activation, and neuronal death (Scheltens et al., 2021). Structural imaging of AD brains observed atrophy of the hippocampus and in later stages, the frontal cortex, areas with high density distribution of CB_1_R (Glass et al., 1997; Biegon & Kerman, 2001; Erkkinen et al., 2018). However, such atrophy is not necessarily correlative of observed deficits in declarative memory and recall (Nelson et al., 2009; Iqbal et al., 2010; Aschenbrenner et al., 2018).

Alterations in CB_1_R expression resultant of AD maintains itself as a point of contention. Studies have reported considerable decreases in CB_1_R expression within post-mortem AD patient brain tissues (Ramírez et al., 2005), particularly those compared to age-matched controls (Solas et al., 2013). Others found no change regarding distribution or expression of CB_1_R within the hippocampus and cortex (Benito et al., 2003; Lee et al., 2010; Mulder et al., 2011; Ahmad et al., 2014). CB_2_R expression has been found to be significantly increased with the accumulation of b-amyloid plaques (Benito et al., 2003; Ramirez et al., 2005; Solas et al., 2013), even in instances where plaque accumulation did not induce cognitive impairment (Solas et al., 2013). Increasing evidence supports a potential contributory role of the serotonergic system in AD. Serotonin 5-HT1A receptors are expressed in the hippocampus and are involved with memory processing and learning (Ögren et al., 2008; Muzerelle et al., 2016; Solís-Guillén et al., 2021).

Preclinical studies have shown marked decreases in 5-HT1A receptor expression across various regions sampled from human AD brains (Bowen et al., 1983; Cross et al., 1984; Lai et al., 2003). 5-HT1A receptors as a target for alleviating cognitive dysfunction has shown promise. Continued research with 5-HT1A receptor antagonists, such as lecozotan, enhanced cognitive function in a rat model of scopolamine induced amnesia (Skirzewski et al., 2010), as well as enhanced cognitive performance in aged rhesus monkeys and reversed cognitive deficits associated with cholinergic lesions in marmosets (Schechter et al., 2005). As partial agonists buspirone and tandospirone both improved AD patient mood and behavior, further research into 5-HT1A modulation, *via* CBD or other cannabinoids with serotonergic activity, may provide promising therapeutics able to alleviate AD associated cognitive dysfunction, behavioral decline and memory impairment (Salzman, 2001; Sato et al., 2007). Indeed, in accordance with CBD’s serotonergic activity profile, CBD has shown utility in animal AD models. Utilizing intracerebroventricular administration of beta-amyloid in mice to simulate cognitive impairment associated with AD, it was shown that intraperitoneal administration of CBD was able to modulate beta-amyloid activation of microglia and restore cognitive function as indicated by decreased latencies in the Morris water maze compared to vehicle controls (Martín-Moreno et al., 2011). Furthermore, THC exhibits neuroprotective effects when administered within a transgenic, beta-amyloid expressing AD mouse model with observed reductions in neuronal loss and reduced accumulation of b-amyloid compared to vehicle controls (Franke et al., 2019).

Recent clinical studies have demonstrated potential therapeutic benefit with nabilone for the treatment of neurodegenerative and neuroinflammatory diseases. In a double-blind, randomized cross-over AD study, markers for oxidative stress and neuroinflammation, such as tumor necrosis factor-α, were decreased following nabilone administration (1–2 mg), indicating a correlative association with reductions in agitation and decreased inflammation markers (Ruthirakuhan et al., 2020).

#### 2.1.2 Parkinson’s Disease

Parkinson’s disease (PD) is the second most common neurodegenerative disease after AD, with an estimated prevalence of 572 per 100,000 among those aged 45 years and older (Marras et al., 2018), and doubling of such instances within the next 2 decades is expected (Dorsey et al., 2018). Characterized by the loss of dopaminergic neurons within the substantia nigra pars compacta, the resulting loss of dopaminergic striatal input nigra leads to the hallmark observable changes of PD. These changes include reductions in motor function such as resting tremor, bradykinesia, postural instability, and rigidity (Davie, 2008) as well as cognitive impairment, mood disorders, and pain sensory disturbances (Erkkinen et al., 2018). While instances of PD are thought to be sporadic, genetic mutations are heavily linked to PD onset, including missense mutations with genes PARK1, PARK2, and PARK7 (Blauwendraat et al., 2020). Proinflammatory signaling is thought to play a role in disease progression as well (for reviews see Wang et al., 2015; Klein and Westenberger, 2012), providing therapeutic potential to cannabinoids and their anti-inflammatory nature.

Research has shown that CB_1_R mRNA expression is decreased within preclinical rat models of toxin induced PD (Silverdale et al., 2001; Walsh et al., 2010) and genetic mouse models of PD (García-Arencibia et al., 2009). However, decreases in mRNA expression observed in genetic mouse models were shown to be reversed in later disease stages with increased CB_1_R mRNA expression (García-Arencibia et al., 2009). Significant reductions in CB_1_R expression within the ventral mesencephalic region are observed in early-stage PD patients compared to healthy controls (Van Laere et al., 2012). Similarly, CB_1_R mRNA was found to be reduced in the caudate nucleus, anterior dorsal putamen, and the external globus pallidus in human post-mortem brain tissues taken from PD patients (Hurley et al., 2003; Van Laere et al., 2012). However, up regulation of both CB_1_R and CB_2_R expression has been observed within the substantia nigra in human post-mortem striatal brain tissues taken from medicated PD patients (Navarrete et al., 2018). Serotonergic systems are affected alongside the dopaminergic denervation associated with PD with observed decreases in serotonin and dopamine concentration in cerebrospinal fluid and human striatum (Tohgi et al., 1992; Kish et al., 2008; Politis & Niccolini, 2015). Using positron emission tomography, it was found that 5-HT1A receptor binding was reduced significantly in PD patients compared to healthy controls, with a significant correlation between reduction in binding and tremor severity (Doder et al., 2003).

Blocking serotonergic signaling with the 5-HT1A agonist buspirone was found to reduce the development of l-DOPA-induced dyskinesia in a 6-hydroxydopamine (6-OHDA) lesion model of PD in rats (Eskow et al., 2007). Using the same 6-OHDA lesion model to induce dopamine depletion associated with PD in rats, it was found that CBD was able to recover dopamine levels when given immediately after lesion induction (García-Arencibia et al., 2009). However, the same study had found that administration of CBD 1 week after the lesion did not affect dopamine levels. Preclinical research studying the effects of THC in PD models have reported potential neuroprotective effects. Utilizing an *in vitro* model of PD with SH-SY5Y cells and PD relevant toxins, THC was shown to have an active neuroprotective effect that mitigated cell death following exposure to toxins that generate free radicals and inhibit mitochondrial function (Carroll et al., 2012). THC treatment within a marmoset PD model was also shown to improve locomotor activity associated with spontaneous exploratory behavior and complex tasks requiring hand-eye coordination (van Vliet et al., 2008). Neuroprotective effects were observed following daily intraperitoneal administration of THC over the course of 2 weeks within a preclinical rat model of toxin induced PD, with THC having reduced dopaminergic loss (Lastres-Becker et al., 2005).

Clinical studies utilizing CBD administration in PD patients have observed a reduction in the occurrence of psychotic symptoms that include sleep disturbances, hallucinations, and delusions (Zuardi et al., 2009), reduced tremor amplitude (de Faria et al., 2020), and an overall observed improvement in patient well-being and motor function (Chagas et al., 2014). A recent clinical trial utilizing CBD (Epidiolex) found similar improvements in PD associated symptoms with patients experiencing good tolerability with no major adverse effects with the 5–25 mg/kg/day dosing schedule (Leehey et al., 2020). A phase II, randomized, placebo-controlled, double-blind study to examine the effectiveness of nabilone to impact non-motor adverse effects related to PD has recently concluded and had found that PD patients given nabilone responded positively to doses up to 1 mg with good tolerability and with no major adverse effects reported (Peball et al., 2020). Clinical assessment surveys and self-scoring methods from this study indicated that patients receiving nabilone experienced improvements to non-motor adverse effects as opposed to the placebo arm, which reported increased disturbances resultant of non-motor adverse effects.

#### 2.1.3 Huntington’s Disease

Huntington’s disease (HD) is a rare genetic neurodegenerative disorder resultant of excessive extension of CAG repeats within the huntingtin gene. Symptoms of HD include alterations in movement, mood, and cognition (see McColgan and Tabrizi, 2018) with an estimated prevalence of approximately 3 cases per 100,000 (Erkkinen et al., 2018). Cardinal features of HD include neuronal death and neuroinflammation in the striatum, globus pallidus, substantia nigra, and cerebral cortex (Hickey and Chesselet, 2003), with advanced stages of HD exhibiting wide-spread neuronal death among the cerebellum, hippocampus, and brain stem (Erkkinen et al., 2018).

Preclinical studies utilizing genetic mouse models of HD observed decreases in CB_1_R mRNA within the striatum, cortex, and hippocampus in initial phases of HD (Denovan-Wright and Robertson, 2000; McCaw et al., 2004; Dowie et al., 2009). In rat preclinical studies utilizing a pharmacological model of HD, both CB_1_R mRNA and protein were decreased in the caudate putamen, basal ganglia, globus pallidus, and substantia nigra (Lastres-Becker et al., 2001, 2002), though administration of substances that increased endocannabinoid activity was found to have activated the decreased population of CB_1_R and improve subject motor function (Lastres-Becker et al., 2002). Utilizing quantitative autoradiography in post-mortem brain tissue sections from HD patients, a significant loss in CB_1_R protein was observed within the globus pallidus, and substantia nigra (Glass et al., 1993; Richfield & Herkenham, 1994). Conversely, reductions in CB_1_R expression have been accompanied by increased expression of CB_2_R among astrocytes and microglia in preclinical rat pharmacological models of HD (Fernández-Ruiz et al., 2007; Basavarajappa et al., 2017). Post-mortem brain tissues from HD patients and transgenic mice models also exhibit increased CB_2_R expression within the caudate putamen as well as in striatal microglia (Palazuelos et al., 2008). Disruption of the serotonergic system has been observed in striatal samples from HD patient brains where serotonin transporter protein was found in increased concentrations compared to age matched healthy controls and early-stage HD brain samples (He et al., 2019). In brains taken from a transgenic HD mouse, binding analyses of 5-HT1A receptors found reduced binding of 5-HT1A receptor agonists among hippocampal and striatal regions of the brain, indicating further disruption of the serotonergic system within HD (Yohrling IV et al., 2002).

Within a rat pharmacological model of HD, THC produced neuroprotective effects, which further suggests that THC may have therapeutic potential, as well as lends additional credence that cannabinoid receptor dysfunction may be involved in HD etiology (Lastres-Becker et al., 2004). In a preclinical study utilizing a rat model of striatal atrophy, CBD administration was able to reverse neurodegeneration following a 5 mg/kg/day dosing schedule over 5 days, and the authors found that these effects were likely the result of the intrinsic antioxidant potential held by CBD (Sagredo et al., 2007).

One small scale pilot study has indicated potential therapeutic capacity of nabilone in HD, having observed improvements to motor skills and participant cognition (Curtis et al., 2009). A case report also observed that medicinal cannabis and nabilone were able to improve patient motor function and cognitive behavior, though no measured responses were taken and reports were anecdotal (Curtis & Rickards, 2006) In contrast, clinical trials have observed either no significant difference in motor function and cognition with nabiximols compared to placebo controls (Lopez-Sendon Moreno et al., 2016), failure to provide symptomatic protection with CBD (Consroe et al., 1991), or significant increases in involuntary movements with nabilone (Müller-Vahl et al., 1999).

#### 2.1.4 Multiple Sclerosis

Multiple sclerosis (MS) is a debilitating neurodegenerative disease largely affecting individuals in early adult life with an increasing prevalence of 1 per 3,000 individuals, or approximately 2.8 million people worldwide (Walton et al., 2020). Characterized pathologically by hallmarks that include inflammation, axonal and neuronal loss, demyelination, and astrocytic gliosis within the brain stem and spinal cord (Goldenberg, 2012; Thompson et al., 2018), MS is physiologically characterized by episodes of sensory and motor impairments driven largely by neurodegeneration.

Preclinical studies utilizing experimental autoimmune encephalomyelitis MS models have observed reduced expression of CB_1_R among the striatum and cortex of rats (Berrendero et al., 2001), and mice deficient in CB_1_R used in experimental autoimmune encephalomyelitis MS models exhibit enhanced neurodegeneration compared to control subjects (Pryce et al., 2003). Similarly, studies utilizing human post-mortem CNS tissue samples have observed increased expression of CB_1_R among cortical neurons, oligodendrocytes, oligodendrocyte precursor cells and macrophages near plaques associated with MS (Benito et al., 2007; Palazuelos et al., 2008). Likewise, CB_2_R receptor expression and density were found to have been increased in MS, particularly in T-lymphocytes, astrocytes, microglia, and macrophages near active plaques (Yiangou et al., 2006; Benito et al., 2007). Utilizing photon emission tomography, patients with MS were found to have lower availability of serotonin transporters throughout the limbic system, a factor that may contribute to the psychiatric symptoms associated with MS, as well as disturbed modulation of the immune system (Hesse et al., 2014). CBD has been shown to provide therapeutic benefits for the treatment of MS, though further research is needed to understand the mechanisms driving such activity. Preclinical studies utilizing experimental autoimmune encephalomyelitis in mice have found that CBD administration ameliorated the severity of MS symptoms when given during disease onset (Kozela et al., 2011), inhibited production of pro-inflammatory cytokines (Giacoppo et al., 2017), and attenuated the infiltration of CD4^+^ T cells and macrophages into the central nervous system (Constantinescu et al., 2011; Giacoppo et al., 2017).

Although nabiximols administration has not been found to improve cognitive function of patients (Rekand, 2014; Lopez-Sendon Moreno et al., 2016; Riva et al., 2019), the THC:CBD spray combination has not been associated with cognitive decline in long-term use (Rekand, 2014), a salient concern, given that long-term THC use has been linked to poor cognitive health (Crean et al., 2011). Compared to placebo controls, early clinical trials demonstrated that nabiximols displayed efficacy in the alleviation of MS-associated spasticity and reduced both spasm number instances and severity during treatment (Collin et al., 2007; Novotna et al., 2011; Wade et al., 2004, 2006). A more recent clinical trial utilizing nabiximols for the treatment of symptoms associated with MS observed superior improvement of MS induced spasticity compared to adjustments in first-line anti-spasticity medication alone (Markova et al., 2019). Similar improvements have been reported with clinical trials utilizing 10 mg dronabinol, where reductions in spasticity and improved ambulation (University of California, Davis, 2018) and modest improvements in pain assessments (Svendsen et al., 2004) were observed. While these studies report good tolerability with no major adverse effects, dronabinol was found to have no improvements on cognitive function and indications of worsening cognitive function with time (University of California, Davis, 2018). Whole cannabis extract was also found to have relieved muscle stiffness experienced by MS patients (Zajicek et al., 2012). Finally, medicinal cannabis was found to have slightly reduced fatigue among MS patients compared to age/sex matched controls with no record of cannabis use (Rudroff, 2020). Though this comparative observational study reports good tolerability as well, there is no mention of total cannabis consumption among patients. These findings are summarized in Table 1.

#### 2.1.5 Amyotrophic Lateral Sclerosis

Amyotrophic lateral sclerosis (ALS) is a neurodegenerative disease with a prevalence between 4.1 and 8.4 per 100,000 persons, characterized by gradual loss of muscle control resultant of increasing muscle weakness and wasting (Longinetti & Fang, 2019). This results in the progressive loss of the ability to chew, swallow, talk and breathe, ultimately leading to death (Zarei et al., 2015). Portions of those with ALS will also experience frontotemporal dementia and changes in behavior and cognition as the disease progresses (Masrori & Damme, 2020). While 90–95% of all ALS cases are sporadic with unknown etiology (Zarei et al., 2015) and pathogenesis is not completely understood, it is thought that mechanisms associated with excitotoxicity, oxidative stress, and neuroinflammation are implicated with ALS onset (Rao & Weiss, 2004; Liu & Wang, 2017; Batra et al., 2019).

Preclinical research utilizing a genetic mouse model of ALS presents conflicting observations regarding CB_1_R. Compared to healthy controls, reductions in spinal cord motor neuron CB_1_R expression has been observed in the early, pre-symptomatic stage in a mouse ALS model, with an elevation of expression observed in the symptomatic stage (Zhao et al., 2008). The authors suggest that this may be indicative of a neuroprotective action compensating for initial losses in CB_1_R, though ultimately, expression of CB_1_R was reduced in end-stage ALS mouse models, suggesting continued declination in neuronal health (Zhao et al., 2008). Another preclinical study utilizing genetic ablation of CB_1_R in a genetic mouse model of ALS observed an extension of life span compared to wild type subjects (Bilsland et al., 2006). However, ablation also resulted in significant motor neuron death and decreased survival of remaining motor neurons. An immunocytochemistry analysis of post-mortem spinal cord tissue taken from ALS patients observed increased CB_2_R immunoreactivity within areas exhibiting neuronal degeneration (Yiangou et al., 2006). Further analyses will be needed to understand the role of cannabinoid receptors in ALS experienced by humans. Motor neurons preferentially affected in ALS are densely innervated by 5-HT expressing neurons; their degeneration may provide the pathological link to the spasticity commonly seen with ALS (Sandyk, 2006; Dentel et al., 2013). In ALS patient brainstem samples, it was found that there was severe degeneration of serotonergic neurons compared to healthy controls (Dentel et al., 2013).

Studies utilizing a transgenic mouse model of ALS have observed that mice treated with THC (Raman et al., 2004; Weydt et al., 2005), experienced delayed disease progression and prolonged survival. An *in vitro* component to one such study found that THC effectively reduced oxidative stress and minimized excitotoxicity within mouse spinal cord cultures (Raman et al., 2004), suggesting that THC possesses potential neuroprotective effects which may be beneficial in treating ALS.

Current clinical research into the use of cannabinoids in ALS is limited, though previously mentioned clinical trials in other neurodegenerative diseases suggest therapeutic potential in ALS. Observations gathered from patient surveys suggest that medicinal cannabis provides therapeutic relief of symptoms of ALS such as pain, spasticity, and excessive drooling, however these observations are limited by the comparatively small number (10% of those surveyed) of those having used cannabis recently at the time of survey (Amtmann et al., 2004). A randomized placebo-controlled clinical study utilizing nabiximols in patients with ALS report reductions in spasticity symptoms with no report of major adverse effects (Riva et al., 2019). Finally, a clinical study investigating the efficacy of THC in mitigating cramping associated with ALS observed no subjective improvement of cramp intensity among ALS patients, though THC was well tolerated with no major adverse effects reported with 10 mg daily oral administrations (Weber et al., 2010). These findings are summarized in Table 1.

#### 2.1.6 Epilepsy

Seizures are the result of abnormal synchronous neuronal excitation within the brain with etiologies including genetic predisposition, injury, brain tumors, and neurodegenerative diseases (Falco-Walter, 2020). The prevalence of epilepsy is between 50.4 to 81.7 per 100,000 persons annually and continues to rise as advances in healthcare lead to increased survivability of traumatic head injuries, stroke, and increased lifespans (GBD 2016 Epilepsy Collaborators, 2019).

As discussed above cannabinoid compounds have exhibited antispastic capacity in a range of neurodegenerative disease states that gives further support to the use of cannabinoids for the treatment of epileptic convulsions. THC-related anticonvulsant activity is likely the result of CB_1_R stimulation. Preclinical evidence suggests that the endogenous cannabinoid system contributes to the regulation of seizure frequency. Mice lacking functional CB_1_R or mice that have genetic alterations in endogenous cannabinoid system activity which lead to decreased CB_1_R tone are characterized to be seizure prone (Clement et al., 2003; Marsicano et al., 2003). THC completely abolished spontaneous seizures within a rat model of epilepsy, while CB_1_R antagonism with SR141716A increased seizure duration and frequency (Wallace et al., 2003). The same study also revealed that CB_1_R expression was significantly increased within epileptic hippocampi (Wallace et al., 2003). CBD-related anticonvulsant activity is likely due to its 5-HT agonist properties, actions at voltage-gated sodium ion channels, as well as its ability to modulate intracellular calcium storage. *In vitro* studies, CBD selectively inhibited aberrant sodium currents in mutated sodium ion channel expressing cells and had no effect on normal sodium channel activity (Patel et al., 2016). In a mouse *ex vivo* epilepsy model CBD pre-exposure blocked aberrant hippocampal nerve firing, and these protective effects were inhibited by either a reduction in serotonin tone, or pharmacologically, by a calcium store antagonist (Maggio et al., 2018).

Clinical research has yielded promising results with the use of cannabinoids for treating epilepsy. However, current interest has been focused largely on CBD due to good tolerability and lack of psychoactive effects (Jones et al., 2010). CBD utilization within clinical trials of treatment-resistant epilepsy (Devinsky et al., 2016; Gaston et al., 2019) and Dravet syndrome (Devinsky et al., 2018, 2019) with clinically approved Epidiolex was found have significantly reduced the occurrence and duration of epileptic seizures. Long term safety and quality of life studies with Epidiolex also indicated that CBD provides effective long-term treatment with good tolerability and improves patient quality of life (Gaston et al., 2019; Laux et al., 2019).

#### 2.1.7 Increasing Prevalence Requires Further Research

The prevalence of neurodegenerative and neurological disease continues to rise globally as improvements in healthcare result in improved survivability of many previously fatal diseases and longer life spans. However, with increases by more than 35% in death rates among those with neurodegenerative diseases within the past 25 years, therapeutics are needed urgently. Many of the pathologies discussed above lack any current clinically approved cures or treatments, with the current extent of our therapies only providing symptomatic relief. With endocannabinoid targets such as CB_1_R and CB_2_R and serotonergic involvement with 5-HT1A, development of cannabinoid-based therapeutics shows promise and further research and development is critical considering our aging population.

### 2.2 Pain

Pathological pain is a substantial component of many chronic illnesses and diseases and can be divided into pain that arises from inflammatory insults, known as inflammatory pain, and pain that is the result of nerve injury, known as neuropathic pain. Both inflammatory and neuropathic pain alter neuronal processing and immune cell function. These alterations ultimately lead to perceived pain with a prevalence of 6.9–10% for neuropathic pain, with much higher estimates for inflammatory pain as it is ubiquitous in many disease states (van Hecke et al., 2014). Diseases with exhibitions of neuropathic pain include diabetes, neurodegeneration, human immunodeficiency virus, and chemotherapy induced peripheral neuropathy. Cancer itself has both inflammatory and neuropathic components that contribute to pain perception, with common cancers such as breast, prostate, kidney, and lung cancers resulting in metastasis to bone that further drive and contribute to pathological pain in cancer (Mantyh, 2014).

#### 2.2.1 Endocannabinoid Targets in Pain

There is continued interest in the endocannabinoid system and its involvement in pain modulation (see Donvito et al., 2018). This is due to the extensive expression of CB_1_R throughout the CNS in pain relevant regions such as afferent nerve fibers (Hohmann et al., 1999; Morisset and Urban, 2001), spinal cord interneurons (Jennings et al., 2001), trigeminal sensory neurons (Price et al., 2003), and neurons within the periaqueductal grey (Mailleux and Vanderhaeghen, 1992). As for the periphery, CB_1_R is observed on peripheral nociceptors (Richardson et al., 1998) and the dorsal root ganglia (Hohmann and Herkenham, 1999). As for CB_2_R, these cannabinoid receptors are expressed in peripheral macrophages and lymphocytes (Munro et al., 1993; Bouaboula et al., 1993; Galiegue et al., 1995) as well as astrocytes, oligodendrocytes, and microglia within the central nervous system (Zhang et al., 2003; Maresz et al., 2005; Beltramo et al., 2006; Racz et al., 2008), suggesting potential mediation of inflammatory pain with cannabinoids. The spatial distribution of CB_1_R and CB_2_R within the CNS are shown in Figure 1. CB_2_R may also be implicated with neuropathic pain, as CB_2_R has observed expression among sensory neurons of the dorsal horn after sciatic nerve section or spinal nerve ligation in rats (Wotherspoon et al., 2005). 5-HT1A receptors are expressed in areas relevant to pain signaling and transmission such as primary afferent neurons, peripheral terminals, astrocytes, oligodendrocytes, and microglia (Björk et al., 1992; Laporte et al., 1995; Granados-Soto et al., 2010; Perrin et al., 2011). The spatial distribution of 5-HT1A receptors within the CNS is shown in Figure 1.

#### 2.2.2 Preclinical Studies in Pain

Preclinical studies assessing CBD for pain relief have painted a promising picture for the cannabinoid. Though as discussed in the next section, expectations should be tempered as the translational efficacy of cannabinoids into humans is unclear. CBD has been found to exert analgesic effects in animal models of neuropathic pain, such as surgically induced nerve injury and chemotherapy induced neuropathy (Harris et al., 2016; Ward et al., 2011, Ward et al., 2014), though effects are dependent on dose and route of administration (Costa et al., 2007; Abraham et al., 2020). While the therapeutic window of THC is limited by its psychoactive side effects, various studies utilizing combinations of THC and CBD have found improved efficacy in low dose administrations to treat pain. In models of either surgically induced nerve injury (Casey et al., 2017; Linher-Melville et al., 2020) or chemotherapy induced neuropathy (King et al., 2017), 1:1 THC + CBD combinations exhibited greater efficacy at low doses that were ineffective with either THC or CBD alone.

#### 2.2.3 Clinical Trials in Pain

Despite these preclinical studies suggesting therapeutic potential of cannabinoids as analgesics, review of recent clinical trials in various pain pathologies suggests an inconclusive viability of cannabinoids as a therapeutic for pain. Within a double-blind placebo-controlled study in MS patients experiencing neuropathic pain, THC (dronabinol), taken orally up to a maximum dose of 15.9 mg over 16 weeks, decreases patient reported pain measurements, though no significant difference from placebo was ever observed (Schimrigk et al., 2017). Studies in safety also found that whole cannabis was able to alleviate chronic pain whilst improving patient quality of life with both acute and long-term administrations (Ware et al., 2010, 2015). An open label, long-term efficacy and safety portion of this clinical trial also observed maintained decreases in patient reported pain intensities with low occurrences of serious adverse effects being reported (Schimrigk et al., 2017). A clinical trial utilizing THC (dronabinol) for pain relief in patients with noncardiac chest pains observed that 10 mg oral administrations improved patient pain thresholds significantly compared to placebo with good tolerability and with no major adverse effects being reported (Malik et al., 2017). Clinical trials utilizing nabiximols have observed pain relief in patients experiencing pain resultant of a range of pathologies including MS (Notcutt et al., 2004; Rog et al., 2005; Kavia et al., 2010), peripheral neuropathy (Nurmikko et al., 2007), and cancer (Johnson et al., 2010; Portenoy et al., 2012; Fallon et al., 2017; Lichtman et al., 2018). A clinical trial with nabiximols in patients experiencing chronic pain associated with late-stage cancer observed a 15.5% improvement among patient reported perceptions of pain (Lichtman et al., 2018). An identical companion study to this clinical trial once again observed similar improvements with nabiximols (Fallon et al., 2017). It should be noted that patient pools utilized cohorts from both the United States and Eastern Europe, with significant improvements in pain relief compared to placebo among American patients and general improvements among Eastern Europeans, though these effects were not significant compared to placebo in Eastern European cohorts (Fallon et al., 2017; Lichtman et al., 2018). It should be noted in these studies the Eastern European cohort was sicker than the American cohort, suggesting that patient selection criteria may have contributed to the divergent study findings. Additionally, nabiximols administration did meet several secondary endpoints associated with quality of life, which, as suggested by the authors, may indicate therapeutic utility in cancer pain as an adjuvant therapeutic with a low opioid dose (Lichtman et al., 2018). In a small double-blinded, placebo-controlled, crossover design clinical study, oral CBD had no effect on muscle damage markers or muscle soreness in exercised untrained men (Cochrane-Snyman et al., 2021). A recent double-blind, placebo controlled clinical study in an emergency room setting found that orally administered CBD was equal to placebo and did not adequately control acute non-traumatic low back pain (Bebee et al., 2021). Although these above clinical studies suggest CBD may not be a frontline analgesic, further clinical studies examining various route of administration and dosing strategies are needed. Additionally, it is unknown if the other compounds in medicinal cannabis may yield enhanced utility of these cannabinoids in clinical pain management settings.

#### 2.2.4 Opioids and Cannabinoids for Pain

The ability of medical cannabis to augment the analgesic potency of opioids without additional enhancement of opioid associated side effects is an area of growing research interest with recent clinical trials yielding mixed results. Clinical pain research suggests that medicinal cannabis or cannabinoids for chronic pain may yield opioid sparing effects, a major consideration given the interest in minimizing opioid use for severe pain to avoid opioid tolerance, dependance risk, and side effects such as somnolence and respiratory depression. Studies utilizing smoked or oral medicinal cannabis among habitual opioid using, chronic pain patient cohorts observed improvements to quality of life, pain, and opioid prescription cessation (Haroutounian et al., 2016; Capano et al., 2020). Indeed, following 6 months of opioid/cannabis cotreatments, prescribed morphine use was found to have dropped significantly compared to baseline usage among patients with chronic pains, with reductions observed earlier at 3 months opioid/cannabis cotreatment (O’Connell et al., 2019). Similar reductions in prescribed, opioid use were also observed over a 21-month period with 83.8% of patients (*N* = 37) reporting reduced prescribed daily opioid dosage and 40.5% of patients ceasing opioid prescriptions altogether (Vigil et al., 2017). A clinical trial utilizing healthy participants with no prior indication of pathological pain had observed that orally administered THC (dronabinol, 5 mg) was not able enhance the analgesic effects of oxycodone in coadministrations and reported an increase in both abuse and impairment related effects associated with opioid use (Babalonis et al., 2019). A similar study in healthy subjects also reported that THC (dronabinol, max 10 mg) had no consistent dose-effect relationship with the opioid agonist hydromorphone in measures of both acute and chronic pain, though significant analgesia in acute pain with hydropmorphone and 2.5 mg dronabinol compared to placebo was observed (Dunn et al., 2021). Additionally, a clinical trial utilizing healthy cannabis smokers found that the combination of oxycodone (2.5 mg) and cannabis (cigarettes, 5.6% THC) was not able to provide analgesia in measures of acute pain and increased abuse-related subjective effects but did increase pain thresholds and tolerance (Cooper et al., 2018). This discrepancy may be due to the contributing pharmacological effects of the other compounds found in cannabis rather than THC alone, though more work is needed to explore the complex pharmacology between cannabinoids and opioids.

#### 2.2.5 Disconnect Between Preclinical and Clinical Research Findings

Pain is a substantial component of many chronic illnesses and diseases that range from cancer to diabetes. With extensive expression of CB_1_R and CB_2_R and 5-HT receptors, cannabinoid compounds have great potential as novel pain therapeutics for pathologies such as chemotherapy induced peripheral neuropathy, cancer, and neurodegenerative diseases. Current preclinical literature shows promise with cannabinoids being able to effectively alleviate pain across different animal pain models. However, clinical research suggests that more work is needed to examine dose, pain indication, and route of administration questions, given that many studies observe general, but not significant, improvements in pain when compared to proven analgesics such as oxycodone and other opioids. Despite this, these studies and others still report improvements in patient reported assessments of pain and quality of life compared to placebo controls. As such, further clinical research is warranted to determine whether cannabinoids can provide effective pain relief alone or as an adjunctive therapeutic in human pain pathologies.

### 2.3 Addiction and Substance Use Disorders

From alcohol to opioids, addiction can occur with a variety of psychoactive drugs and consists of use disorders characterized by heavy consumption, loss of intake control, and withdrawal experiences (Zou et al., 2017). Most of these addictive substances can result in elevations of extracellular dopamine that, with time, downregulate the expression of dopamine receptors and negatively affect dopaminergic neurons, like those found in the mesocorticolimbic pathway (Wise & Robble, 2020).

#### 2.3.1 Endocannabinoid Targets in Addiction and Substance Use Disorders

In addition to dopaminergic receptors, CB_1_R are expressed in abundance throughout this pathway in brain regions involved in reward signaling such as the ventral tegmental area, nucleus accumbens, amygdala, pre-frontal cortex, and hippocampus (Oleson et al., 2021). Preclinical studies utilizing either CB_1_R antagonists or deletion of the receptor have observed reduced motivation for the consumption and self-administration of ethanol in rat and mouse alcohol dependence models (Gallate et al., 2004; Thanos et al., 2005; Femenía et al., 2010). Rimonabant, the CB_1_R inverse agonist/functional CB_1_R antagonist, was shown to reduce conditioned place preference and self-administration of alcohol (Arnone et al., 1997), heroin (De Vries et al., 2003), and nicotine (Cohen et al., 2005; Robinson et al., 2018). While serious psychiatric side effects such as anxiety, depression, and suicide ideation have prevented rimonabant from passing clinical trials (Manzanares et al., 2018), it supports the notion that CB_1_R antagonism may allow for the attenuation of substance use disorders. CB_2_R in substance use disorders may also provide a potential target with cannabinoid therapeutics as research suggests their potential role in modulating behaviors associated with addiction (Onaivi et al., 2008b; Agudelo et al., 2013; Galaj et al., 2020). Similarly, given the role that the serotonergic system plays in both motivational and reinforcement processes, serotonergic modulation may provide a solution to alleviating substance use disorders (Müller & Homberg, 2015; Yagishita, 2020). Research shows that extracellular serotonin is acutely increased following administration of morphine (Tao & Auerbach, 1994; Fadda et al., 2005) and alcohol (Yoshimoto et al., 1992; Bare et al., 1998; Thielen et al., 2002). Additionally, chronic administration of psychoactive substances such as morphine, ethanol, and cocaine have been found to reduce the basal levels of extracellular serotonin within the brain, potentially resulting in increased sensitivity (Pelloux et al., 2012; Müller & Homberg, 2015). Preclinical research focused on 5-HT1A receptor modulation in the context of drug reward and addictive behaviors found CBD decreased morphine-induced reward facilitation in an operant behavioral paradigm within rats that was mediated through 5-HT1A receptor activation in the dorsal raphe nucleus (Katsidoni et al., 2013). Though further studies are required, current research has provided proof of concept regarding the treatment of drug dependency and use disorders with cannabinoids, suggesting their use as an alternative or co-adjuvant therapeutic.

#### 2.3.2 Alcohol Use Disorder

US Food and Drug Administration approval for drugs in the treatment of alcohol use disorder has not occurred since 2004 with the approval of acamprosate. Preclinical research has shown that CBD may hold particular promise as an alcohol use disorder therapeutic. Activity at CB_2_R may provide for an initial target in therapeutic development with cannabinoids. An early study utilizing the CB_2_R agonist, JWH 015 in stressed mice observed enhanced alcohol preference compared to controls (Onaivi et al., 2008a). Upregulation of CB_2_R was also observed in dendritic cells from patients with alcohol abuse disorders (Agudelo et al., 2013). Preclinical studies utilizing mice in the two-bottle choice paradigm and oral ethanol self-administration demonstrated that systemic CBD administration significantly reduced both ethanol consumption and preference, suggesting that CBD can reduce the motivational properties of ethanol (Viudez-Martínez et al., 2018). The same study found that CBD administration prevented relapse in oral ethanol self-administration (Viudez-Martínez et al., 2018). Contrary to the potential benefits observed with CBD, THC has been found to reinstate alcohol seeking behavior in abstinent rats (McGregor et al., 2005). Utilizing a beer (4.5% ethanol v/v) self-administration paradigm, the study observed that intraperitoneal THC administration significantly reinstated responding previously reinforced with beer. However, both sucrose trained subjects and beer trained subjects had self-administration responses reinstated with THC administration (McGregor et al., 2005).

#### 2.3.3 Opioid Use Disorder

Preclinical studies with cannabinoids as a therapeutic for opioid use disorders are largely motivated by the neurobiological interactions between the cannabinoid and opioid systems (Rodríguez et al., 2001; Schoffelmeer et al., 2006). A preclinical study in rats utilizing a self-administration, drug-seeking behavior model found that CBD inhibited reinstatement of cue-induced heroin seeking behavior, though such effects were not observed with drug seeking behavior initiated by a priming dose of heroin (Ren et al., 2009). A similar study utilizing a conditioned place preference paradigm in mice with morphine treatment found that CBD decreased the establishment of opioid reward as indicated by an attenuation of morphine place preference (Markos et al., 2018). In contrast, some research suggests that THC may not be a viable therapeutic for treating opioid use disorders, though use in treating withdrawal symptoms show promise. It has been observed that subjects pre-exposed in adolescence shown marked opiate sensitivity with higher consumption of heroin and upward shifts in self-administration acquisition (Ellgren et al., 2007). Similar studies observing the effects of systemic THC administration in cannabinoid-opioid system interactions have reported similar results with enhanced opioid intake in operant behavioral studies (Vela et al., 1998; Solinas et al., 2004). THC may not be without therapeutic opioid use disorder utility. Current research, though limited, demonstrates that systemic THC administration inhibits symptoms (jumping, rearing, wet shakes, diarrhea) associated with naloxone-induced opioid withdrawal (Hine et al., 1975; Bhargava, 1976).

#### 2.3.4 Tobacco Use Disorder

While currently limited, there is increasing evidence that cannabinoid compounds are able to modulate tobacco use disorder. However, some studies are seemingly contradictory, and have left the exact therapeutic utility of cannabinoids for the treatment of tobacco use disorder unclear. A small-scale pilot study in treatment-seeking smokers had found that use of a CBD inhaler resulted in reduced self-reported smoking compared to placebo treatment over a 7-day period, although cravings for cigarettes remained unchanged (Morgan et al., 2013). Acute administration of THC has been found to attenuate the somatic and motivational manifestations of nicotine withdrawal in mice, though it is unlikely a result of the compensatory changes on CB_1_R density following chronic nicotine exposure (Balerio et al., 2004). A similar study assessing nicotine and THC coadministration in mice found enhancement of both the expression of nicotine withdrawal symptoms and nicotine induced conditioned place preference (Valjent et al., 2002). Further research will be required to elucidate any potential therapeutics for nicotine use disorders.

#### 2.3.5 Cocaine Use Disorder

With no currently approved therapeutics for psychostimulant addiction, the use of cannabinoids as a treatment for cocaine addiction has garnered interest despite a small body of literature. Recent preclinical work with CBD administration in mice observed reduced CB_1_R expression within the nucleus accumbens with simultaneous increases in CB_2_R expression (Calpe-López et al., 2019). These results are intriguing and raise the possibility that although CBD may not act directly *via* CB_1_R or CB_2_R based upon binding affinity, it may alter cannabinoid receptor tone. An earlier study utilizing JWH133, a CB_2_R agonist, found that CB_2_R agonism was able to dose dependently inhibit cocaine-enhanced locomotion and cocaine self-administration in mice (Xi et al., 2011). Such effects were not observed in CB_2_R knockout mice and were blocked with AM630, a CB_2_R antagonist, suggesting a role for CB_2_R in modulating cocaine-induced rewarding and locomotor enhancing effects (Xi et al., 2011). A similar study conducted last year in mice also reported similar benefits, as CBD prevented behavioral alterations associated with cocaine addiction that included locomotor stimulation and memory deficits related to cocaine withdrawal (Ledesma et al., 2021). Finally, in a rat cocaine self-administration model, it was observed that CBD reduced cocaine self-administration, and these effects are blocked following CB_2_R and 5-HT1A antagonist administration (Galaj et al., 2020).

#### 2.3.6 Considerations of Cannabis Dependence and Therapeutic Capacity

As mentioned earlier, the two primary constituents of cannabis are THC and CBD, with THC having psychoactive properties and marked effects on dopamine release like other drugs of addiction. Indeed, clinical research shows that acute administration of THC does elicit dopamine release within the striatum (Bossong et al., 2015; Bloomfield et al., 2016), with such effects being dose dependent. Chronic cannabis use is also associated with increased risk of substance use disorder development and development of withdrawal behaviors that include irritability, anxiety, depression, fever, and tremors (Katz et al., 2014; Volkow et al., 2014). Furthermore, utilization of THC in anxiolytic therapies is limited due to psychoactive sequelae, risk of abuse, and anxiogenic effects (Kayser et al., 2020b; García-Gutiérrez et al., 2020). While the therapeutic window of THC is limited by its psychoactive side effects, various studies utilizing combinations of THC and CBD have found improved efficacy in low dose administrations to treat pain, anxiety, and depression. Although the negative attributes of cannabis are largely attributed to THC, as described above, CBD continues to draw attention as an anxiolytic and analgesic. The minor components of cannabis may also prove to be beneficial in either the selective development of whole cannabis therapeutics or as isolated cannabinoid compound mixtures. This rationale is due to the discovery that cannabis terpenoids and minor phytocannabinoids exhibit therapeutic capacity in a variety of pathologies, including epilepsy, neurodegenerative disease, and traumatic brain injuries (Russo & Marcu, 2017; Russo, 2018).

### 2.4 Anxiety Disorders

Early epidemiological studies observing the prevalence of mood disorders had found that anxiety disorders are highly prevalent within the United States (Weissman, 1988; Stein et al., 2017). Anxiety disorders to date are maintained as the most common mood-related disorders (Vos et al., 2015; Penninx et al., 2021) both within the United States and worldwide. Psychological symptoms of common anxiety disorders include frequent and prolonged states of amplified fear and/or anxiety (Giacobbe & Flint, 2018).

#### 2.4.1 Endocannabinoid Targets in Anxiety

Brain regions relevant in feelings of anxiety and fear include the prefrontal cortex, hippocampus, amygdala, hypothalamic nuclei, and the bed nucleus of the stria terminalis (Lafenetre et al., 2007), regions with notable expression of neuronal CB_1_R. Additionally, CB_2_R present within the periphery and the CNS, have been implicated in both anxiety disorders and anxiety regulation (Garcia-Gutierrez and Manzanares, 2011; Liu et al., 2017; Patel et al., 2017). Like CB_1_R, 5-HT1A receptors are present in high densities throughout the CNS in areas associated with emotional control and anxiety, including regions such as the hippocampus, amygdala, and cerebral cortex (Mestikawy et al., 1991; Wang et al., 2009; Marcinkiewcz et al., 2016). While their direct role in anxiety onset is unclear, the contribution of the serotonergic system is evident as 5-HT1A receptor knockout mice exhibit increased anxiety-like behavior in assays such as the elevated-plus maze and open-field test, both of which provide face and predictive validity in human models of anxiety (Hirshfeld et al., 1992; Graeff et al., 1998; Lesch, 2005).

#### 2.4.2 Cannabinoid Consideration for Anxiety

Primary first line pharmacotherapeutics for the treatment of anxiety are serotonergic, which include selective serotonin reuptake inhibitors (SSRIs) and azapirones like buspirone. These therapeutics are generally well tolerated with short-term adverse effects that include nausea, diarrhea, and constipation. However, more problematic adverse effects include sexual dysfunction (Jing & Straw-Wilson, 2016), suicide ideation in pediatric patients (Hammell et al., 2016), and serotonin syndrome (Volpi-Abadie et al., 2013) with SSRIs and the development of buspirone induced movement disorders (Rissardo & Caprara, 2020). While the utilization of THC in anxiolytic therapies is limited due to psychoactive sequelae, risk of abuse, and anxiogenic effects (Kayser et al., 2020b; García-Gutiérrez et al., 2020), CBD continues to draw increasing attention in its use as an anxiolytic as work continues in developing therapeutics that can mimic the beneficial effects of current first line anxiety therapeutics while having improved side effect profiles over SSRIs and buspirone. CBD has been indicated as a potential treatment of a range of anxiety disorders that include both generalized anxiety disorder (GAD) and social anxiety disorder (SAD) as well as the excessive anxiety associated with post-traumatic stress disorder (PTSD) and obsessive-compulsive disorder (OCD) (Micale et al., 2013; Blessing et al., 2015).

#### 2.4.3 Preclinical Studies in Anxiety

Preclinical literature regarding CBD in rodent models of generalized anxiety suggest CBD’s efficacy in minimizing anxiety associated behaviors relevant in GAD, SAD, PTSD, and OCD. Studies utilizing CBD in elevated plus and elevated T mazes with rodents have observed anxiolytic effects following both acute systemic administration (Campos et al., 2012; Campos et al., 2013a; Campos et al., 2013b) and acute local administrations in areas such as the amygdala central nucleus (Hsiao et al., 2012), bed nucleus of the stria terminalis (Gomes et al., 2011), and the intra-dorsal periaqueductal gray (Soares et al., 2010). Anxiolytic effects of CBD in these models are presented as a bell-shaped dose-response curve, with anxiolytic effects generally observed at moderate doses; 2.5–10.0 mg/kg in rats (Guimarães et al., 1990), 1 and 10 mg/kg in mice (Onaivi et al., 1990). Chronic administrations of CBD have also been found to produce anxiolytic effects in mice with the open-field test (Long et al., 2010), though contrasting results from a later study show that chronic CBD had no such effect in the elevated plus maze (Schiavon et al., 2016). Despite these mixed results and considering current preclinical evidence, use of CBD as an anxiolytic appears favorable with an improved side effect profile and no risk of anxiogenic effects (Garakani et al., 2020).

#### 2.4.4 Clinical Trials in Anxiety

Secondary outcomes of clinical trials utilizing dronabinol (Malik et al., 2017), nabilone (John Redmond et al., 2008), and oral titrations of THC (Attal et al., 2004) all have reported general improvements to patient anxiety alongside their primary outcomes on pain relief. Studies assessing cannabinoid/opioid cotreatments also observed improvements to patient quality of life with secondary outcomes looking at measures of anxiety (Haroutounian et al., 2016; Capano et al., 2020). Direct assessments of patient anxiety provide clinical evidence that suggests that CBD has potential as a treatment for anxiety disorders, though such studies have generally focused on acute administrations utilizing small subject sizes often in healthy patients (Blessing et al., 2015; García-Gutiérrez et al., 2020). Clinical studies utilizing the simulation public speaking test had found that acute oral administration of CBD capsules reduced subjective (visual analog mood scale) and physiological (blood pressure, heart rate) measures of stress in healthy patients (Zuardi et al., 2009; Bergamaschi et al., 2011). Of these studies, a treatment naïve patient group with SAD was given CBD and had also exhibited indications of reduced anxiety, both subjective and physiological (Bergamaschi et al., 2011). Clinical studies assessing the anxiolytic properties of cannabinoids in PTSD and chronic pain pathologies have also observed general improvements in patients having consumed whole cannabis products (Greer et al., 2014; Bonn-Miller et al., 2021) or CBD alone (Elms et al., 2019) as indicated by lowered scores in clinician administered posttraumatic stress scales and PTSD checklists which assess emotional response and cognitive function. Instances of self-reported anxiety associated with OCD were found to have been no different than placebo after administration of cannabis high in either CBD (0.4% THC/10.4% CBD) or THC (7.0% THC/0.18% CBD) (Kayser et al., 2020a). Another retrospective clinical study had found that general anxiety experienced by patients was reduced following continued CBD administration using the Hamilton anxiety rating scale, though it should be noted that this study utilized open-label treatment for patients without a comparison group (Shannon et al., 2019). These studies support the potential for CBD as a treatment for anxiety disorders, especially when paired with preclinical findings. However, larger clinical trials assessing both acute and chronic dosing in additional anxiety disorders are needed.

#### 2.4.5 CBD, But Not THC for Anxiolytic Development

Anxiety disorders are highly prevalent within the United States and is maintained as the most common mood-related disorder worldwide. First line therapeutics for the treatment of anxiety include SSRIs and buspirone and while generally tolerated, these therapeutics are associated with problematic adverse effects that include sexual dysfunction and suicide ideation in pediatric patients. Therefore, development of cannabinoid-based anxiolytics would provide a potentially safer alternative to current therapies. It should be noted though that the psychoactive components of cannabis, such as THC, are generally anxiogenic at higher doses and while clinical research has indicated anxiolytic effects at low doses, THC alone seems to have fallen out of favor in anxiolytic development. With indications from both preclinical and clinical research, CBD may prove to be an effective cannabinoid in relieving anxiety in patients and further development of cannabinoid-based anxiolytics is warranted.

## 3 Considerations for Cannabinoid Administration and Formulation

Cannabinoids exhibit particular characteristics that must be considered for both compound formulation and routes of administration as the pharmacokinetics and effects observed are heavily dependent on these (Lucas et al., 2018). Cannabinoids such as THC exhibit high lipophilicity, low aqueous solubility, and susceptibility to degradation *via* light, heat, and auto-oxidation (Grotenhermen, 2003). Interest in cannabinoid formulation, delivery strategies, and utilization of optimal routes of administration continues to grow in parallel with interests in the use of cannabinoids for potential therapeutic applications. Formulation strategies have been developed to overcome challenges brought upon by characteristics such as high lipophilicity in other compounds, though these strategies require testing in cannabinoids to determine if they would provide favorable pharmacokinetic improvements in items such as distribution and bioavailability (Kumari et al., 2010; Allen and Cullis, 2013; Dengler et al., 2013).

### 3.1 Oral Administration

Current clinically approved cannabinoids such as nabilone, dronabinol, and cannabidiol (Epidiolex) utilize oral administration and is the most prevalent route of administration for therapeutic applications. In the case of dronabinol, an exciting opportunity presents itself where direct comparisons can be made between the capsule and liquid formulation of the cannabinoid regarding efficacy. Companion clinical trials aiming to assess potential differences between these capsule and liquid formulations had observed a large, though insignificant, difference in dronabinol absorption times with 4.25 mg liquid formulations being superior to 5 mg capsules (Parikh et al., 2016; Oh et al., 2017). However, peak serum concentration was higher for both dronabinol and its metabolite, 11-OH-Δ9-THC, with the capsule formulation (Parikh et al., 2016; Oh et al., 2017). This disparity among formulations may likely be the result of hydrophobic drugs being less bioavailable when delivered in oil-based formulations (MacGregor et al., 1997). Indeed, both THC and CBD oral administrations in sesame oil exhibit poor bioavailability, as low as 6% in humans (Agurell et al., 1981; Huestis, 2005), likely resulting from variable absorption and extensive first pass metabolism (Lucas et al., 2018). Utilization of a self-emulsifying drug delivery system (SEDDS) could provide a more desirable endpoint for compound bioavailability as the mixing oils, surfactants, solvents, and other excipients can improve the oral bioavailability of lipophilic compounds. SEDDS may provide a viable solution to the challenges brought upon by the inherent lipophilicity of cannabinoids as patents filed by Murty Pharmaceuticals show a growing body of research that supports this drug delivery system (Murty and Murty, 2012). A recent clinical study utilizing a SEDDS-CBD oral administration (standardized to 25 mg) in healthy volunteers has observed significant improvements across pharmacokinetic parameters, including increased CBD plasma values, enhanced bioavailability, and fast absorption with no safety concerns being noted (Knaub et al., 2019). Ultimately, these oral formulations could provide symptomatic relief over prolonged periods (Lucas et al., 2018), making them suitable for continued administrations for the chronic symptomatic relief.

### 3.2 Nasal and Oral Mucosal Administrations

Alternative routes of administration can provide methods of circumventing variable absorption and extensive first pass metabolism. Both the oral mucosa and nasal cavity provide attractive targets for alternative routes of administration due to thin layering coupled with extensive vascularization. An assessment report conducted by the Australian Department of Health’s Therapeutic Goods Administration for nabiximols surmised that oromucosal formulations of nabiximols are rapidly absorbed, resulting in higher plasma concentrations of THC and CBD compared to oral formulations (Therapeutic Goods Administration, 2013). Though administration *via* the nasal mucosal membrane provides favorable absorption rates, current formulations are not as attractive given patient reluctance, formulation safety concerns, and nasal spray particle size (Therapeutic Goods Administration, 2013). It is likely that these issues associated with nasal administration has resulted in few recent developments regarding intranasal formulations (Bryson & Sharma, 2017; Bruni et al., 2018). Current oromucosal formulations of cannabinoids are therefore preferable in providing rapid, potentially therapeutic effects in a manner that is comfortable to patients and is more likely to be self-administered.

### 3.3 Pulmonary Administration

Among the possible routes of administration utilized for cannabinoids, pulmonary administration of cannabis is likely the most well-known route of administration among the general population. Like nasal and oral mucosa, pulmonary administration is highly effective given the high bioavailability, rapid onset, and avoidance of first-pass metabolism this route provides (Grotenhermen, 2003). However, critical issues associated with both intrapatient and interpatient variability arise given the inherent variability with pulmonary administration without the use of standardized methods. These include variations in inhalation depth, irritation or discomfort, technique and experience, and pharmacokinetic parameters such as maximum plasma concentration (Ohlsson et al., 1982; Hunault et al., 2010; Solowij et al., 2014; Lucas et al., 2018). Factors such as these could ultimately affect the efficacy of inhaled cannabis or cannabinoids and should be considered with concerns of dosing frequency and of side effects such as intoxication and cognitive function with psychoactive components like THC. Much interest surrounds the development of a standardized system or device that can deliver a metered dose of inhaled cannabinoid as a result. Comparatively, cannabinoid vaporization has grown in popularity due to ease of use and relative safety compared traditional combustion methods, such as with cannabis cigarettes (Gieringer et al., 2004). However, a standardized methodology has yet to be developed to account for sources of variability such as inhalation depth, though some have presented method proposals (Solowij et al., 2014; Lanz et al., 2016) and metered inhalation device patents (Davidson et al., 2018). A clinical study utilizing this metered inhaler observed that the product was able to administer consistent doses (15.1 ± 0.1 mg) of cannabis (19.9% THC, 0.1% CBD, 0.2% cannabinol) that provided effective neuropathic pain relief in patients (Eisenberg et al., 2014).

### 3.4 Topical Applications

Like mucosal and pulmonary administration, topical administration of cannabinoids provides an avoidance of first-pass metabolism, steady administration over time, and consistent dosing. However, due to the hydrophobicity of cannabinoids, diffusion across the skin is limited and such topical formulations require enhancements to permeation (Challapalli & Stinchcomb, 2002; Lodzki et al., 2003). Current applications for topical cannabinoids, though almost exclusively CBD, range from treating inflammatory dermatological disorders to localized pain relief among instances of arthritis and joint pain, though continued research suggests potential benefit in neuropathic pains (Baswan et al., 2020; D’Andre et al., 2021; Hammell et al., 2016; Xu et al., 2020). Similar to other aspects of cannabinoids as potential therapeutics, advances in formulation and optimization of administration drives further interest research into cannabinoids, though more work is needed.

### 3.5 Standardized Oral Administrations Over Non Standardized Pulmonary Administrations

Pharmacokinetic characteristics of cannabinoids is dependent on route of administration. While clinically approved cannabinoids such as dronabinol utilize oral administrations, general consumption of cannabis is primarily pulmonary with inhalation of cannabis smoke. However, unlike these oral administrations with a standardized formulation and administration method, pulmonary administrations of cannabis introduce numerous variables that could introduce interpatient and interpatient variability. These same factors can also directly affect the kinetics of the cannabinoid. While standardization and development of inhalation devices are being developed, current oral administrations, either through ingestion or through the oral mucosa, provide ease of use among patients and standardization to ensure consistency in dosing.

## 4 Discussion

The field of cannabinoid research continues to experience advancements as interest in therapeutic applications continuously grows, whether that be from urgent needs in replacement therapeutics or the development of a therapeutic the first of its kind. This review of current and past studies finds that preclinical research indicates therapeutic potential for cannabis, THC, and CBD mediated through either CB_1_R, CB_2_R, 5-HT1A, or a variable combination of these receptors. Clinical research utilizing cannabinoids within instances of neurodegenerative disease, pain, addiction, and anxiety suggest both tolerability and therapeutic potential either alone or in combination with current therapeutics. However, preclinical literature dominates, and additional clinical studies are required to clarify these therapeutic indications before definitive declarations can be made. Further advancement of cannabinoids to the clinical setting is dependent on these clinical trials. There still exists a wide gap between the purported and anecdotal medicinal cannabis uses and specific therapeutic indications irrefutably supported by strong scientific evidence. One possible explanation for this disparity may lie in the complex pharmacological nature of cannabis. Although this review focuses on THC and CBD, there are over 100 different compounds in cannabis including minor cannabinoids, cannabis terpenoids, and phytocannabinoids which have additional pharmacological and biological activity. Additional work in the field of medicinal cannabis to identify the exact composition of studied strains, including minor cannabinoid and terpenoid profiles and concentrations, which can vary dramatically between different cannabis strains, is sparse. This information is desperately needed within the field to study interactive effects between minor cannabinoids, terpenoids, as well as THC and CBD. Indeed, it may be that the interactive pharmacological profiles of minor cannabinoids and terpenoids may underlie at least some of the purported medicinal cannabis benefits that have so far been elusive to definitively confirm.

## Abbreviations

AD, alzheimer’s disease; ALS, amyotrophic lateral sclerosis; CBD, cannabidiol; CB1R, cannabinoid 1 receptor; CB2R, cannabinoid 2 receptor; CNS, central nervous system; GAD, generalized anxiety disorder; HD, Huntington’s disease; MS, multiple sclerosis; OCD, obsessive-compulsive disorder; PD, Parkinson’s disease; PTSD, post-traumatic stress disorder; 5-HT1A, serotonin 1a; SEDDS, Self-emulsifying drug delivery system; SSRIs, selective serotonin reuptake inhibitors; SAD, social anxiety disorder; THC, Δ9-tetrahydrocannabinol.
